# Bone marrow mesenchymal stem cells do not enhance intra-synovial tendon healing despite engraftment and homing to niches within the synovium

**DOI:** 10.1186/s13287-018-0900-7

**Published:** 2018-06-19

**Authors:** Mohammad R. Khan, Jayesh Dudhia, Frederic H. David, Roberta De Godoy, Vedika. Mehra, Gillian Hughes, Stephanie G. Dakin, Andrew J. Carr, Allen E. Goodship, Roger K. W. Smith

**Affiliations:** 1 Royal Veterinary College, Hawkshead Lane, Hatfield, AL9 7TA UK; 2 Present address: Writtle Agricultural College, Lordship Road, Chelmsford, CM1 3RR UK; 3 Botnar Research Centre Institute of Musculoskeletal Sciences, University of Oxford, Oxford, OX3 7LD UK; 4 UCL Institute of Orthopaedics and Musculoskeletal Science, Stanmore, HA7 4LP UK

**Keywords:** Intra-synovial tendon injury, Deep digital flexor tendon, Mesenchymal stem cells, Ovine, Magnetic iron-oxide nanoparticles

## Abstract

**Background:**

Intra-synovial tendon injuries display poor healing, which often results in reduced functionality and pain. A lack of effective therapeutic options has led to experimental approaches to augment natural tendon repair with autologous mesenchymal stem cells (MSCs) although the effects of the intra-synovial environment on the distribution, engraftment and functionality of implanted MSCs is not known. This study utilised a novel sheep model which, although in an anatomically different location, more accurately mimics the mechanical and synovial environment of the human rotator cuff, to determine the effects of intra-synovial implantation of MSCs.

**Methods:**

A lesion was made in the lateral border of the lateral branch of the ovine deep digital flexor tendon within the digital sheath and 2 weeks later 5 million autologous bone marrow MSCs were injected under ultrasound guidance into the digital sheath. Tendons were recovered post mortem at 1 day, and 1–2, 4, 12 and 24 weeks after MSC injection. For the 1-day and 1–2-week groups, MSCs labelled with fluorescent-conjugated magnetic iron-oxide nanoparticles (MIONs) were tracked with MRI, histology and flow cytometry. The 4, 12 and 24-week groups were implanted with non-labelled cells and compared with saline-injected controls for healing.

**Results:**

The MSCs displayed no reduced viability in vitro to an uptake of 20.0 ± 4.6 pg MIONs per cell, which was detectable by MRI at minimal density of ~ 3 × 10^4^ cells. Treated limbs indicated cellular distribution throughout the tendon synovial sheath but restricted to the synovial tissues, with no MSCs detected in the tendon or surgical lesion.

The lesion was associated with negligible morbidity with minimal inflammation post surgery. Evaluation of both treated and control lesions showed no evidence of healing of the lesion at 4, 12 and 24 weeks on gross and histological examination.

**Conclusions:**

Unlike other laboratory animal models of tendon injury, this novel model mimics the failed tendon healing seen clinically intra-synovially. Importantly, however, implanted stem cells exhibited homing to synovium niches where they survived for at least 14 days. This phenomenon could be utilised in the development of novel physical or biological approaches to enhance localisation of cells in augmenting intra-synovial tendon repair.

## Background

Tendons connect skeletal muscle to bone and throughout their length can be either extra-synovial or intra-synovial; the latter being when the tendon is surrounded by a synovial tendon sheath that allows almost frictionless movement of the tendon where it wraps around bony prominences. Intra-synovial tendon disease is not only a common orthopaedic condition in humans but also affects domestic mammals that suffer naturally occurring tendon injuries, such as horses. In humans, the rotator cuff tendons of the shoulder undergo an age-related degenerative condition which is the third most common musculoskeletal complaint that affects 5–30% of adults [1, 2]. Forty to 50% of patients in the UK consult their general practitioners for shoulder pain [3, 4] which poses a significant socio-economic and treatment challenge. The presence of a rotator cuff tear has been found to correlate with poorer shoulder scores [5] and can impair the ability to work or perform household tasks and result in increased time off from work [6, 7].

This injury involves the fused tendons of supraspinatus, infraspinatus, teres minor and subscapularis that are located between the subacromial bursa and the scapulohumeral joint, and frequently tears enter one or both of these synovial cavities. Once in communication with the synovial environment, spontaneous healing is frustratingly poor and the release of tendon extracellular matrix components induces a strong inflammatory response [8]. ‘Failed’ healing is thought to be a consequence of the poorly understood adverse effects of synovial fluid on tendon healing [9] and the lack of reparative cells, usually derived from peritendinous sources [10] such as the paratenon which is absent within intra-synovial portions of tendons.

Current treatment strategies for this disease frequently deliver ineffective results in terms of functionality and/or pain. They are routinely treated with injections of a mixture of local anaesthetic and corticosteroid. Evidence would suggest that these injections are no more effective than physiotherapy and result in 40–50% of patients having persistent or recurrent symptoms after 1 year [11]. Surgery, often contemplated for persistent cases, can be unsuccessful in the long term with reported failure rates between 13 and 68% [12–15]. Not surprisingly, larger tears are associated with higher incidences of re-rupture, and re-rupture has been shown to correlate with poorer outcomes [16, 17]. There is much interest in the use of the newer biological treatments that can potentially enhance surgical treatments, such as the use of platelet-rich plasma (PRP) which aims to deliver high levels of anabolic growth factors to the area. However, in recently published randomised controlled trials, there has not been a consistently significant long-term improvement with the use of PRP either by intra-tendinous injection [18, 19] or by augmentation of surgical repairs [20–28], although the re-tear rate may be reduced [25, 29]. There is therefore a strong unmet clinical need to restore tendon integrity and avoid surgery.

Intra-synovial deep digital flexor tendon tears in the horse occur within the digital sheath where the tendon affected is under compression and the tear communicates with a synovial environment [30], identical to rotator cuff tears in humans, even though it is in a different anatomical location. Conservative management is also usually unsuccessful with affected horses remaining lame and suffering repeated bleeding and inflammation within the tendon sheath. Surgical treatments consist of tenoscopic debridement of the torn tissue but, similar to rotator cuff disease, have had disappointing outcomes [30, 31].

The application of mesenchymal stromal cells (MSCs) for tendon healing has demonstrated efficacy in the treatment of experimentally created tendon injuries in a number of experimental small animal models [32–34]. However, these small animal models show effective healing different from large animal models and human clinical disease [35]. MSCs have been used clinically for the treatment of naturally occurring extra-synovial overstrain injuries in horses and appear to improve healing [36–38] but require intra-tendinous administration into a contained lesion which is not the case in intra-synovial tendon tears. Therefore, from our knowledge of naturally occurring intra-synovial tendon tears in horses, we have developed a large animal experimental model utilising minimally invasive surgery which better replicates the features of intra-synovial tendon disease to assess the effectiveness of MSC therapy. The present study therefore evaluated the distribution and localisation of autologous adult bone marrow-derived MSCs after intra-synovial implantation in a sheep deep digital flexor tendon model of tendon injury. A quadruped’s shoulder is anatomically and functionally different from the human shoulder such that current small experimental models using shoulder tendons, which are neither compressed nor intra-synovial, are inappropriate to investigate clinically relevant intra-synovial healing [35]. The sheep DDFT mimics the functional similarities between the horse DDFT within a tendon synovial sheath and rotator cuff tears in the human, these being a similar biomechanical environment (compression), an intra-synovial location and failed healing with persistent pain, even after tenoscopic debridement [39]. MSCs were initially labelled with fluorescence probe conjugated superparamagnetic iron-oxide nanoparticles (MIONs) to enable MRI, histological and flow cytometry-based evaluations of cellular distribution after intra-synovial injection, and later the outcome of tendon healing was assessed grossly and histologically compared to saline injected controls at 4, 12 and 24 weeks post implantation.

## Methods

### Animals

The study was conducted along the guidelines set by the ethics committee at the Royal Veterinary College and under Home Office licence (no. PPL70/6105). Healthy adult female sheep of the English mule breed were used for this study, with the following treatments (see Table 1):

- Group 1: 1 day (*n* = 2), treated with MION-labelled MSCs.
- Group 2: 1–2 weeks (*n* = 4), three sheep treated with MION-labelled MSCs and one sheep with Dulbecco’s phosphate buffered saline (PBS).
- Group 3: 4 weeks (*n* = 12), six sheep treated with non-labelled MSCs and six sheep with PBS.
- Group 4: 12 weeks (*n* = 16), eight sheep treated with non-labelled MSCs and eight sheep with PBS.
- Group 5: 24 weeks (*n* = 16), eight sheep treated with non-labelled MSCs and eight sheep with PBS.

**Table 1:** Experimental design of animal study

Group	Time point	Number of sheep			Surgery	BM aspiration	MION-labelled MSCs	Non-labelled MSCs	Gross assessment	MRI	Flow cytometery	Histological evaluation
		Control (PBS)	Treated (MSC)	Total								
1	1 day	0	2	2	●	●	●		●	●	●	●
2	1–2 week	1	3	4	●	●	●		●	●		●
3	4 weeks	6	6	12	●	●		●	●			●
4	12 weeks	8	8	16	●	●		●	●			●
5	24 weeks	8	8	16	●	●		●	●			●

*BM* bone marrow, *MION* magnetic iron-oxide nanoparticle, *MRI* magnetic resonance imaging, *MSC* mesenchymal stem cell, *PBS* phosphate buffered saline, dots indicate procedures performed for each group

Smaller numbers of animals were used in Groups 1 and 2 because these were initial studies designed to assess the retention and distribution of labelled MSCs after implantation at a short time point (Group 1, physical spread and retention of cells within the tendon sheath) and after longer time points (Group 2, engraftment). In addition, the hind limbs of two sheep from Group 1 were amputated immediately after euthanasia and assessed for the distribution of MION-labelled MSCs 1 h after implantation into the digital flexor tendon sheath.

### Tendon injury model

The experimental procedure consisted of three stages: creation of a surgical lesion in the deep digital flexor tendon; intra-synovial injection of MSCs or saline 2 weeks after lesion creation; and euthanasia for analytical examination by MRI (for labelled MSCs) and gross and histological evaluation of healing (for non-labelled MSCs) at varying time points after cell injection.

#### Surgical lesion creation

Each sheep was initially anaesthetised using 2% Rompun (xylazine) (Bayer Healthcare) and Ketaset (ketamine) (Fort Dodge Animal Health) at doses of 0.1 and 2 mg/kg of body mass, respectively, and Hypnovel (midazolam) (Roche) at a flat rate of 2.5 mg. General anaesthesia was maintained with isoflurane gas (IsoFlo; Abbott Labs) at 2% of inhaled air. The sheep were maintained in left lateral recumbency.

Bone marrow aspirates were taken from the iliac crest of the right pelvis as described previously [40]. Briefly, approximately 10 ml bone marrow (BM) was aspirated using an 11G Jamshidi needle (CareFusion, USA) into syringes containing 100 IU heparin (Multiparin; CP Pharmaceuticals, Wrexham, UK), which was then transferred into 5 ml of RPMI-1640 (Sigma-Aldrich) on ice for ≤ 3 h prior to isolation of MSCs.

The right forelimb hair was clipped and the circumference of the distal limb above and below the metacarpophalangeal joint was measured. An Esmarch bandage was then applied to the limb terminating proximal to the carpus. A metal rod was inserted into the Esmarch bandage dorsally and taped to the foot to keep the metacarpophalangeal joint in a neutral (straight) position. The tape was used to entirely cover the foot. After aseptic preparation, the limb was draped to expose only the palmarolateral aspect of the metacarpophalangeal joint.

The digital sheath was distended with 2–3 ml saline via a 23G needle inserted in the proximal digital sheath and dorsal to the flexor tendons. Distension of the collateral pouch immediately distal to the proximal sesamoid bones confirmed accurate placement of the needle. A No. 11 scalpel blade was used to create a small (2–3 mm) portal into the digital sheath immediately distal to the proximal sesamoid bone. An arthroscopic sleeve and blunt-ended obturator (Karl Storz GmbH & Co. KG, Tuttlingen, Germany) was then inserted into this portal and gently guided through the fetlock canal. The obturator was removed and replaced with a 2.4-mm 30° forward-angled arthroscope (Karl Storz). The light cable, camera and fluid line were connected and the proximal extent of the sheath was visualised.

**Fig. 1:**
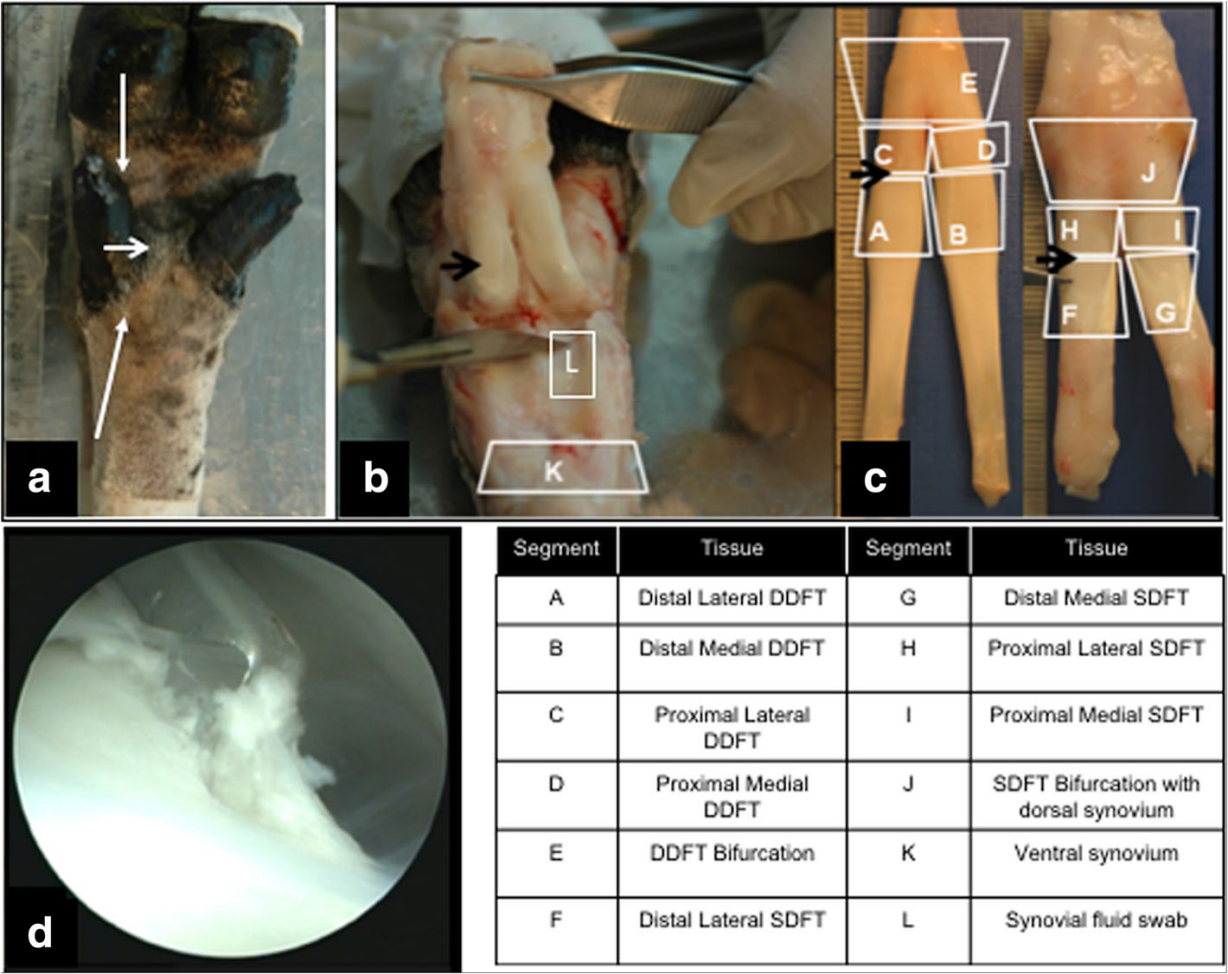
A hook knife was used to create a consistent lesion in lateral DDFT using a tenoscopic approach. Positions of instrument portals marked on intact forelimb by long arrows proximal (hook knife portal) and distal (tenoscope) to ergots (**a**). Relative position of lesion under sesamoid bone shown by short arrows in (a) and (b) at euthanasia. Dissected tendon sheath (comprised of DDFT and SDFT complex) reflected back to expose lesion within normal dorsal opening in SDFT (arrow in **b**). Scalpel blade positioned between tendons and sesamoid bones. Separated tendons shown with DDFT on left and SDFT on right (**c**). Prolapsed fibres from DDFT seen in tenoscope image during surgical generation of lesion, resembling naturally occurring tendon tears (**d**). Tissues divided into 10 segments with an additional segment taken proximal to the sesamoid bones. Identities are elaborated in the accompanying table. DDFT deep digital flexor tendon, SDFT superficial digital flexor tendon

The arthroscope was positioned in the proximal sheath and the site of an instrument portal in the proximal sheath dorsal to the flexor tendons was established using a 23G needle. The portal was created with a No. 11 scalpel and then a 2-mm hook knife (ECTRA II disposable triangle knife; Smith and Nephew, UK) was introduced through the portal and positioned at the distal end of the region of the deep digital flexor tendon not covered by the superficial digital flexor tendon (Fig. 1a–c). The knife was inserted into the deep digital flexor tendon and drawn to the proximal limit of the opening in the superficial digital flexor tendon. The knife was moved repeatedly proximally and distally to ensure an even incision with prolapsed fibres, resembling clinical disease (Fig. 1d). Care was taken to avoid cutting the adjacent superficial digital flexor tendon.

The portals were closed with single simple interrupted sutures of 2-0 monofilament nylon and the area covered in a sterile non-adherent dressing and bandage before recovery from general anaesthesia in a barn.

#### MSC implantation

MSCs were implanted at 2 weeks after creation of the lesion because of the time required to expand the MSCs from the bone marrow aspirate, and this allowed the wound in the synovial sheath to heal to prevent loss of cells through leakage. After induction of general anaesthesia as before, the circumference of the distal limb above and below the metacarpophalangeal joint was again measured. The proximal part of the tendon sheath was prepared aseptically and a 23G arterial catheter was introduced into the lateral compartment of the sheath using ultrasound guidance from the palmar aspect of the limb. The catheter was positioned immediately deep to the flexor tendons and the stylet withdrawn before 5 million MSCs in 1 ml of PBS were injected into the sheath of the treatment group and an identical volume of PBS injected into the control group. Accurate placement was identified by the presence of echogenic air bubbles present inside the sheath cavity after the injection. A bandage was applied to the limb and the sheep allowed to recover from general anaesthesia.

The sheep received two fentanyl (75 μg/h) patches (Durogesic) 12 h prior to surgery followed by a second pair 60 h post surgery, which were followed by 0.6 mg buprenorphine (Vetergesic) 72 h after application of the second pair of fentanyl patches. The sheep were housed in individual pens for 1 week and then group housed with free exercise. Lameness was assessed subjectively on a daily basis throughout the experiment.

#### Euthanasia and post-mortem analysis

Sheep were euthanased using an overdose of 20% pentobarbital at a dose of 0.7 mg/kg (mean volume of 40 ml/animal) at 1 day, 1–2 weeks after implantation of labelled MSCs, and at 4, 12 and 24 weeks after implantation of non-labelled MSCs. The forelimb circumference below and above the metacarpophalangeal joint was determined as a measure of local inflammation before the forelimbs were disarticulated at the carpus for evaluation by MRI (for labelled MSCs) and later dissected for gross and histological examination of the tendons and digital sheath.

### MRI of sheep forelimbs

Isolated forelimbs were scanned from the distal aspect of the digits to the mid metacarpal region with a 1.5 T MR scanner (Philips Intera 1.5 T Pulsar System; Philips Medical Systems, UK). Images were acquired in the transverse plane with a 61 mm × 61 mm field of view, 0.55 mm slice thickness and 0.51 mm × 0.51 mm × 0.55 mm voxel size. Gradient echo sequences (flip angle 15°, TR = 30 ms) were chosen over turbo spin echo sequences in order to maximise sensitivity to the susceptibility artefact generated by the MION particles, resulting in signal void. In order to help differentiate the hypointense signal generated by MION particles from other causes of signal void, such as scar tissue (or other fibrous tissue such as tendons), a dual echo technique (short TE = 7.2 ms and long TE2 = 17.4 ms) was used. This provided spatially matching image series in which the susceptibility artefact associated with MION would increase in size in the long TE images when compared to the short TE images, while the size of other causes of signal void should not be affected.

### Gross evaluation of tendons

Amputated forelimbs were dissected to access the tendon sheath and the digital flexor tendon complex to separate the overlying SDFT from the DDFT. Six gross parameters were evaluated in the 4, 12 and 24-week groups and given a binary score. These parameters were: signs of inflammatory reaction around the digital flexor tendon complex (abnormal discolouration); increased presence of synovial fluid in the sheath using a fine capillary tube; lesion visibility; closure of the lesion either by the presence of tissue covering the lesion and/or the failure of the lesion to open when the tendon was bent; presence of prolapsed fibres at the lesion site; and the presence of adhesions (fibrous attachment between the DDFT and the SDFT).

### Histological evaluation of MION-labelled MSC distribution

Tendons were dissected into 12 segments (Fig. 1), of which seven were fixed in 4% PBS buffered formaldehyde for up to 72 h, followed by dehydration and embedding in paraffin (Fig. 1d, e). Sections for histology were cut on a microtome (Leica RM2125RTF) with N35HR blades (Feather, Osaka, Japan) at 8 μm thickness for staining with H&E (Sigma-Aldrich) according to the supplier’s recommendations.

### Flow cytometry for the assessment of MION-labelled MSCs

Four limbs were analysed with flow cytometry for the distribution of 5 × 10^6^ MION-labelled MSCs after 1 h (two uninjured hindlimbs from two euthanased sheep) and two forelimbs 24 h after implantation. After MRI, hindlimbs were dissected as already described and segmented as shown in Fig. 1 with the accompanying table identifying the histologically evaluated segments from A to L. The seven segments selected for formaldehyde fixation and paraffin embedding were divided into two equal parts. One was used for histology, while the other was digested as a whole to release cells that may have engrafted the tissue surfaces using 1 mg/ml collagenase (type VIII; Sigma-Aldrich) and 1 mg/ml dispase (Gibco, Life Technologies) for 18 h in growth medium in standard tissue culture conditions (37 °C in humidified air and 5% CO_2_). Proteolytic enzymes were deactivated with an equal volume of growth medium and tissue-derived cells isolated and fixed with 4% paraformaldehyde in PBS for 1 h at room temperature. Cells were then washed three times and serially analysed for red fluorescence emissions on a flow cytometer (BD Facs Calibur). Data were analysed with FlowJo version 10.

### Culture and characterisation of MSCs

#### Cell culture

MSCs were isolated from bone marrow aspirates as described previously [40]. Briefly, 10 ml of bone marrow aspirate in RPMI-1640 was combined with an equal volume of Minimal Essential Medium Alpha (α-MEM; Gibco, Life Technologies) supplemented with 15% (v/v) foetal bone serum (FBS; Gibco) and 1% (v/v) of antibiotics (penicillin/streptomycin; Gibco) and seeded in a 75-cm^2^ tissue culture flask (Nunc, Thermo Fisher Scientific, UK) for 24 h in standard tissue culture conditions. The supernatant was discarded and non-adherent cells removed by washing in PBS (Life Technologies) and adherent cells cultured for 7 days. Cells were then passaged and further expanded by re-seeding at a density of 1000 cells/cm^2^ in 175-cm^2^ tissue culture flasks (Nunc) in α-MEM medium for 7 days.

For cell tracking experiments, adherent MSCs were labelled with Molday ION Rhodamine-B conjugated superparamagnetic iron-oxide nanoparticles (BioPal, USA) according to the manufacturer’s guidelines. Briefly, the growth medium of flasks with cells at 70–80% confluence was supplemented with MIONs to a final concentration of 25 μg/ml and incubated for 24 h. Labelling was compared with supplementation of protamine sulphate (Sigma, UK) to a final concentration of 100 μg/ml in an attempt to enhance MION labelling. Cultures were then washed twice with PBS to remove free MIONs and the cells detached by trypsin (Sigma, UK).

A total of 5 × 10^6^ labelled cells suspended in 1 ml PBS were implanted via catheter into the digital flexor tendon sheath as already described. The 4, 12 and 24-week sheep groups received the same dose of unlabelled cells.

#### Microscopy of MION-labelled cells

Confocal microscopy was used to visualise MION uptake by seeding 5 × 10^4^ labelled cells on tissue culture-treated chamber slides (Nunc, Thermo Fisher Scientific, UK) for 24 h. Samples were fixed in 2% paraformaldehyde (Fischer Scientific) in PBS (Gibco) and overlaid with DAPI supplemented mounting medium (Vector Laboratories) for confocal microscopy (Leica 710 confocal microscope).

#### Quantification of MION uptake by MSCs

Particle uptake was quantified by fluorometric analysis of MION-conjugated Rhodamine B. Labelled MSCs suspended in PBS were serially diluted (1:2) with an upper limit of 3.65 × 10^5^ cells in opaque 96-well plates (Nunc). A standard curve was established by serial dilution of MION particles in PBS with an upper limit of 25 μg/ml. Fluorescence emission intensities were obtained at 647 nm in a spectrophotometer (Infinite M200 PRO fluorometer; Tecan, UK). Background-corrected readings of the diluted cells were interpolated from the MION particle standard curve to obtain the quantity of MIONs per cell.

#### Trilineage differentiation

Osteogenic medium comprised Dulbecco’s modified Eagle’s medium (DMEM) low glucose supplemented with 5% FBS, 1% penicillin/streptomycin (Gibco), 10 nM dexamethasone, 5 mM β-glycerolphosphate and 50 μM ascorbate (all Sigma Aldrich). DMEM low glucose without osteogenic supplements was used as a control. Cells were seeded in six-well plates in triplicate at a density of 5000 cells/cm^2^ in control growth and osteogenic media for 21 days with bi-weekly medium changes. Cultures were fixed in 2% paraformaldehyde (Sigma Aldrich) for 20 min before being stained with a solution of 2% Alizarin Red S in water pH 4.3 (Sigma Aldrich).

Adipogenic medium comprised DMEM low glucose supplemented with 10% lot-selected FBS, 1% antibiotics (all Gibco), 0.5 μM 3-isobutyl-1-methyl xanthine (IBMX), 0.5 μM dexamethasone and 50 μM indomethacin (all Sigma). Cells were cultured in 24-well plates in triplicate for control and adipogenic media with bi-weekly medium changes for 21 days. Cultures were washed with PBS and fixed in 2% paraformaldehyde for 20 min before being stained with Oil Red O (Sigma).

Chondrogenic differentiation was conducted according to Solchaga et al. [41]. Approximately 2 × 10^5^–3 × 10^5^ cells in growth medium were pelleted by centrifuging at 400 × *g* for 5 min in 15-ml tubes (Nunc) and the medium replaced with chondrogenic differentiation medium, which was composed of DMEM high glucose (Gibco) with 10% (v/v) insulin–transferrin–selenium (ITS+; BD Biosciences), 100 nM dexamethasone, 1 μM ascorbate-2-phosphate, 1% sodium pyruvate and 10 ng/ml TGF-β3 (R&D Systems). Pellets were maintained for 21 days with bi-weekly medium changes, and then fixed in 2% paraformaldehyde before being processed for paraffin embedding and sectioned to stain with H&E, alcian blue and safranin-O.

### Statistical analyses

The data were subjected to Student’s *t* test or one-way analysis of variance (ANOVA) for significance analysis (*p* < 0.05) using GraphPad Prism 6.02. The data were from at least three independent biological samples and expressed as the mean ± SD.

## Results

### Assessment of clinical parameters post surgery

All surgical procedures were undertaken without complications and animals displayed normal recovery without visible lameness or distal limb swelling. A mild increase in limb circumference above the metacarpophalangeal joint was seen post surgery which reduced after MSC injection, although this was not statistically significant (data not shown). No significant change in body mass was observed during the surgery in either group.

### Characteristics of ovine bone marrow MSCs

**Fig. 2:**
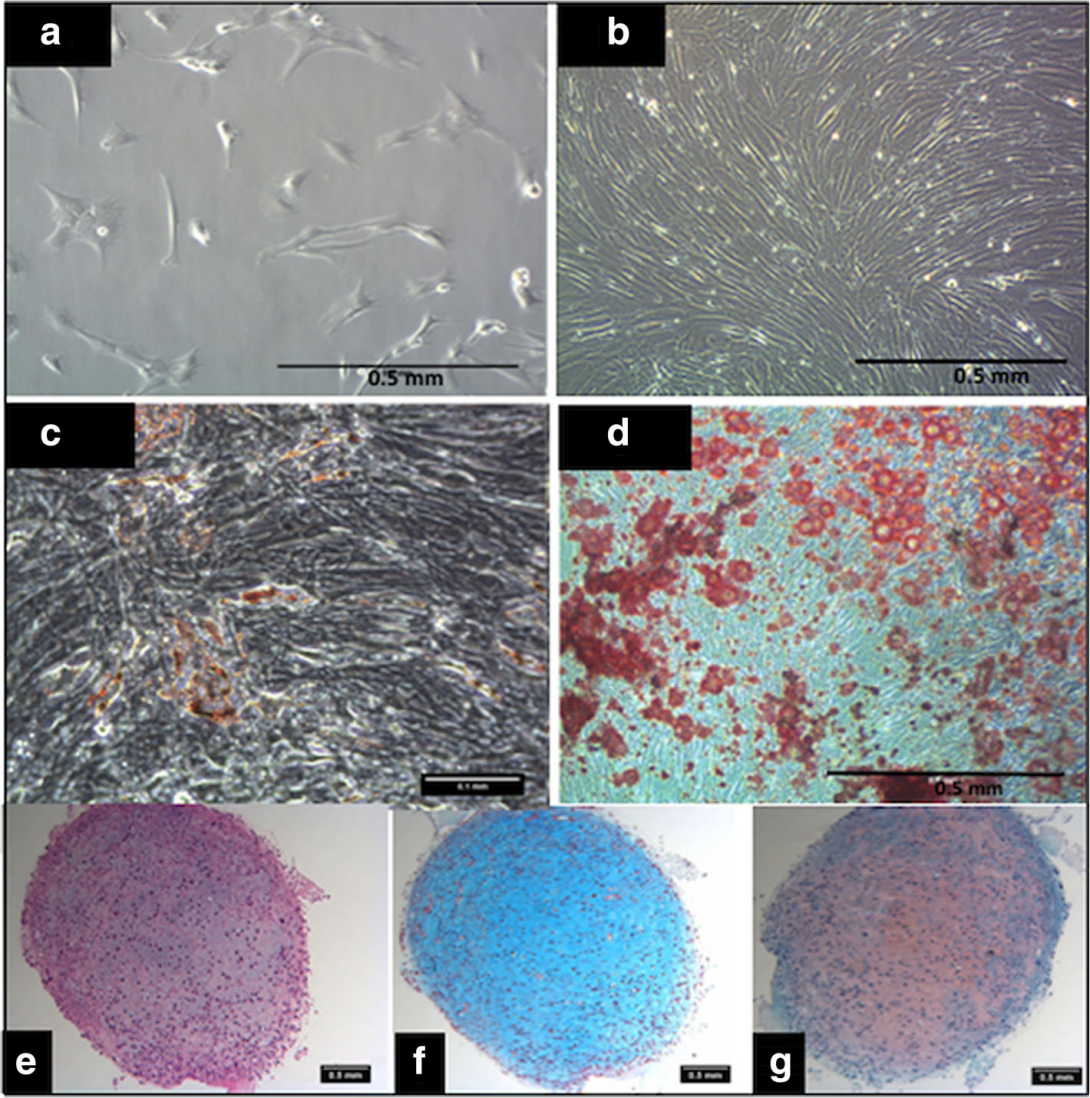
Plastic adherent spindle-shaped cells from bone marrow aspirates (**a**) formed confluent monolayers by 7 days (**b**). Adipogenic Oil Red O-stained MSCs after 14 days of in-vitro differentiation (**c**). Osteogenic differentiation stained with Alizarin Red S staining (**d**). Chondrogenic differentiation with H&E (**e**), alcian blue (**f**) and safranin-O (**g**) staining

BM MSCs displayed a spindle-shaped morphology and formed confluent monolayers (Fig. 2a, b). In CFU-F assays, the MSCs formed colonies when seeded at clonogenic densities with a mean efficiency of 25% (data not shown) and exhibited adipogenic (Fig. 2c), osteogenic (Fig. 2d) and chondrogenic (Fig. 2e–g) differentiation.

### Effect of MION labelling on MSC growth and trilineage potential

**Fig. 3:**
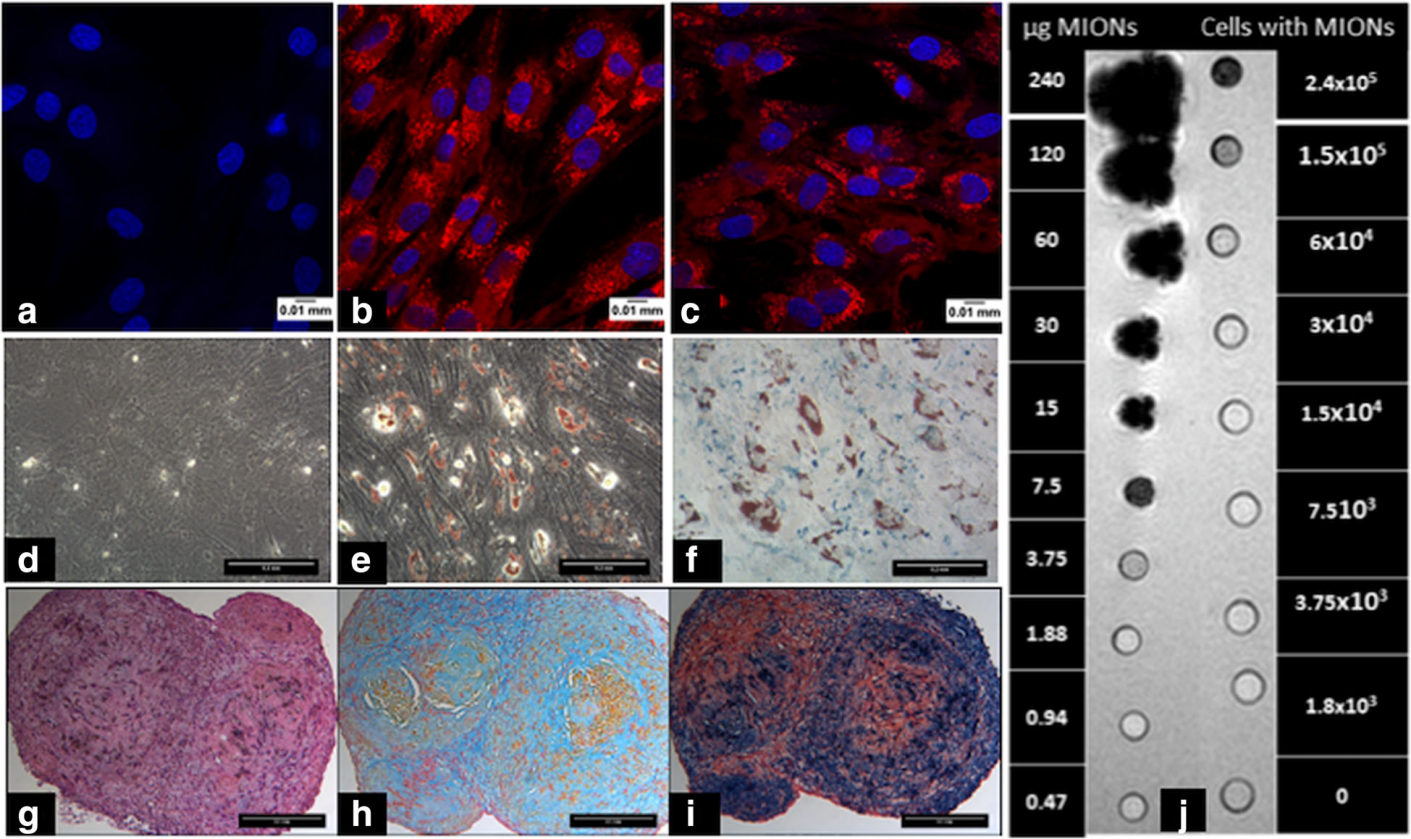
Confocal micrographs of unlabelled MSCs (**a**) and MION-labelled MSCs (**b**) (blue DAPI, red Rhodamine B). Labelled MSCs after 7 days in culture showing persistence of MIONs (**c**). Adipogenic differentiation of MSCs with Oil Red O staining with control cells (**d**) and induced cells (**e**) along with Prussian blue stain (**f**). H&E staining of a chondrogenic pellet of MION-labelled MSCs (**g**) that also stained positively for alcian blue (**h**) and Prussian blue stain (**i**). MR imaging of MION-particles and MION-labelled cells embedded as a bolus in 1% agarose gel to assess sensitivity of detection (**j**). MION magnetic iron-oxide nanoparticle

Confocal microscopy of MSCs consistently demonstrated the successful uptake of MIONs in all samples (Fig. 3a, b) which was not enhanced by the addition of protamine sulphate. MION label was observed to persist in cells cultured for five population doublings over the course of 7 days (Fig. 3c) with no significant difference in the population doubling time between the unlabelled cells (32.29 ± 1.72 h) and labelled cells (34.26 ± 2.98 h, *n* = 3). The MSCs were also not affected in their ability to differentiate in vitro into adipogenic and chondrogenic lineages (Fig. 3d–i) but they did not demonstrate osteogenic differentiation. Fluorometric analyses of MION content revealed 20.04 ± 4.58 pg iron per cell, which conferred a sensitivity of 3 × 10^4^ cells in bolus with MRI (Fig. 3j).

### Tracking of MSCs by MRI

**Fig. 4:**
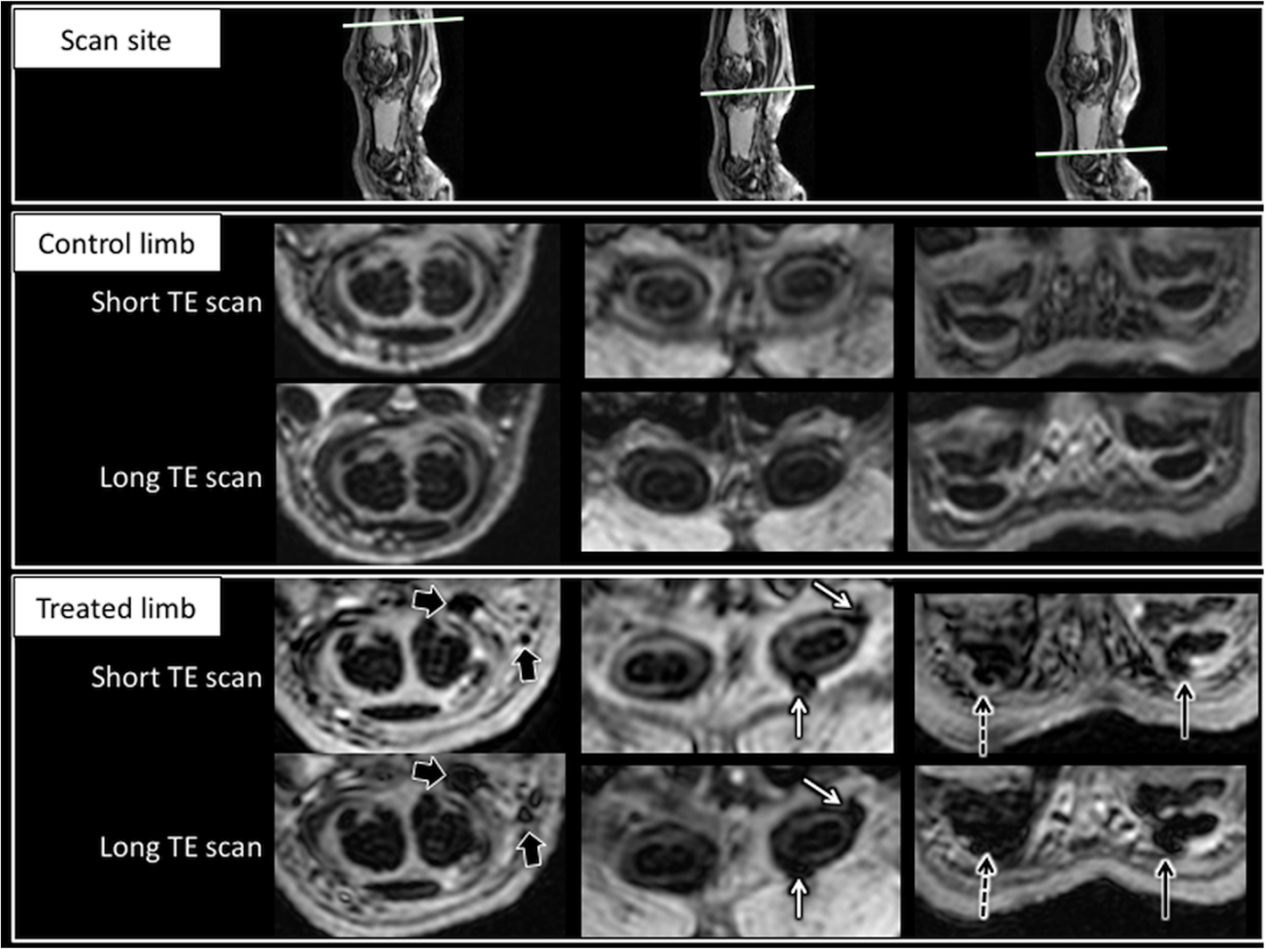
MRI of distal aspect of forelimb. Top row: scan site on a sagittal image. Second and third rows: control limb. Fourth and fifth rows: treated limb. Second and fourth rows: short TE scan. Third and fifth rows: long TE scan. Left columns: images obtained proximal to proximal sesamoid bones. Middle columns: images obtained just distal to proximal sesamoid bones. Right columns: images obtained along distal aspect of proximal phalanx. Susceptibility artefacts (confirmed as such due to larger size on long TE images, compatible with presence of MION particles) identified at multiple sites in treated limb, but not in control limb. Proximally, susceptibility artefact outside tendon sheath (large black arrows) in region of injection site. Just distal to proximal sesamoid bones, susceptibility artefact identified at several sites in lateral tendon sheath (solid narrow white arrows). Distally, susceptibility artefact identified in lateral (solid narrow black arrow) and medial tendon sheath (dashed narrow black arrow). TE echo time

The patterns of localisation were similar in all distal forelimbs injected with MION-labelled MSCs. Areas of signal void (larger on the long TE images) compatible with susceptibility artefacts generated by MIONs were identified in the treated limbs. These predominantly followed a random distribution in the region of the synovial membrane of the tendon sheath (Fig. 4). The tendon lesion was not identified on MRI. Susceptibility artefacts were also identified in the region of the surgical portals (including in PBS control limbs). In some cases, signal voids were identified close to where the tendon lesion had been created, but the limited spatial resolution of MRI and the lack of background signal from the tendons prevented determining whether the origin of the susceptibility artefact was located within the tendon or in the adjacent synovial lining. However, the signal near the tendinous lesion coincided with the histological localisation of MIONs in the adjacent synovium.

### Gross parameters of the tendon synovial sheath and lesion

**Fig. 5:**
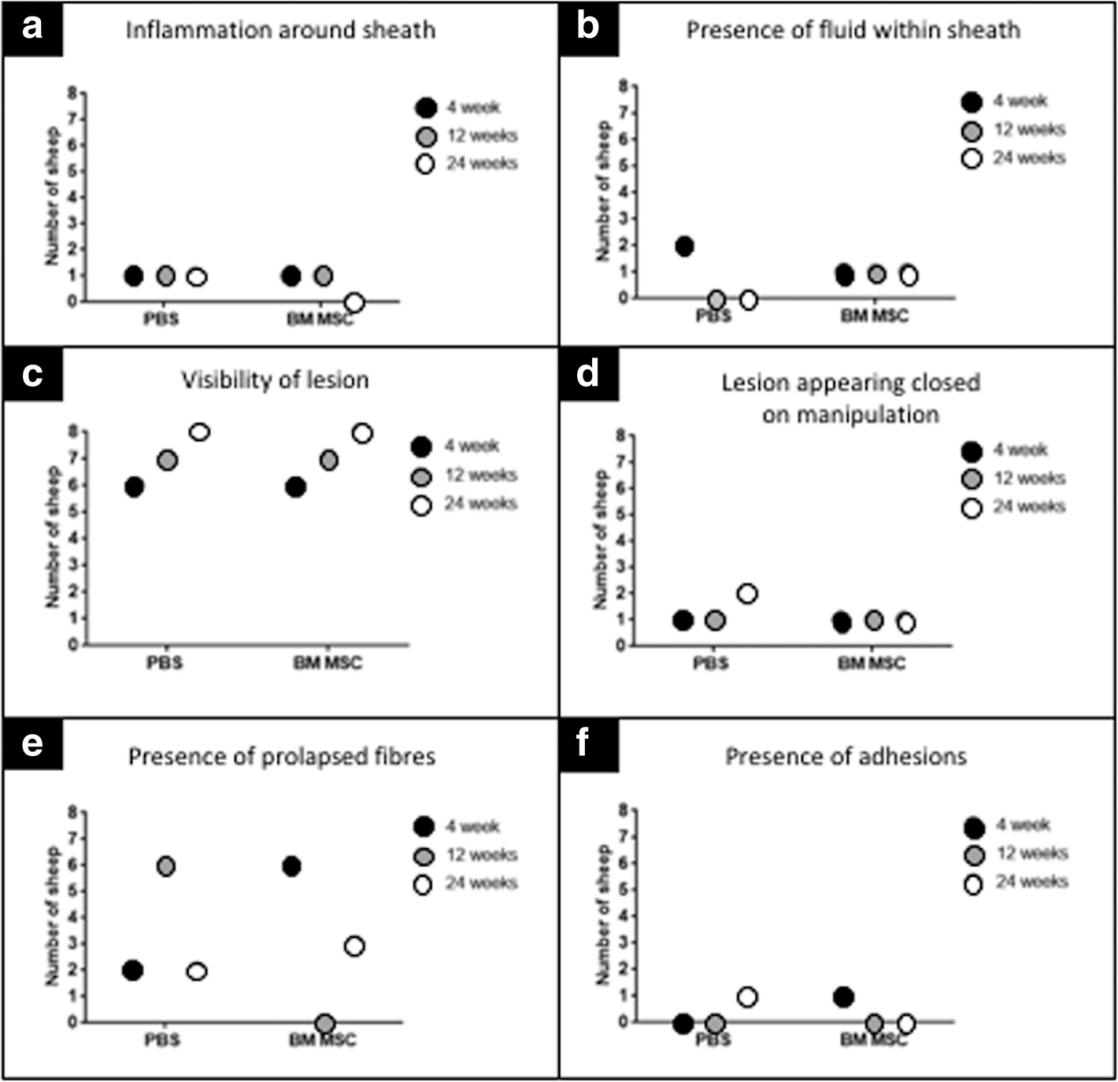
Gross parameters recorded during dissection process indicate only a few animals to have visible inflammation (**a**) with low quantities of fluid within sheath (**b**). Lesion macroscopically visible in most sheep at 4, 12 and 24 weeks (**c**). Dissected tendon gently manipulated by bending in axial plane to determine whether two surfaces of lesion could be separated as a macroscopic sign of healing. At least one sheep in each group showed closure (**d**) and at least one sheep had overlying fibrous tissue but with presence of prolapsed fibres less consistent (**e**). Adhesions were only visible in one sheep (control) at 24 weeks and one sheep (MSC treated) at 4 weeks (**f**). BM MSC bone marrow mesenchymal stem cell, PBS phosphate buffered saline

There was minimal inflammation of the digital flexor tendon sheath. Inflammation was evident in only one sheep in all groups treated with PBS and one sheep at only 4 and 12 weeks treated with MSCs (Fig. 5a). Fluid was visible in two PBS-treated sheep at 4 weeks and one MSC-treated sheep from each of the three time points (Fig. 5b). The lesion was macroscopically visible in all sheep at the 24-week time point (Fig. 5c) and at least one sheep in each group appeared to be closed upon gentle manipulation (Fig. 5d). However, the lesion had overlying fibrous tissue in at least one sheep from both treatments at all time points (Fig. 5e). Prolapsed fibres were visible in 2/6, 6/8 and 2/8 of the PBS-treated sheep at all three time points compared to 6/6 and 3/8 sheep treated with MSCs at 4 and 24 weeks (Fig. 5e). The MSC-treated sheep at 12 weeks displayed no prolapsed fibres (Fig. 5e). Only one sheep in the PBS-treated group and one sheep in the MSC-treated group were found to have adhesions between the DDFT and SDFT (Fig. 5f).

### Histological examination of the SDFT and DDFT for MSCs

**Fig. 6:**
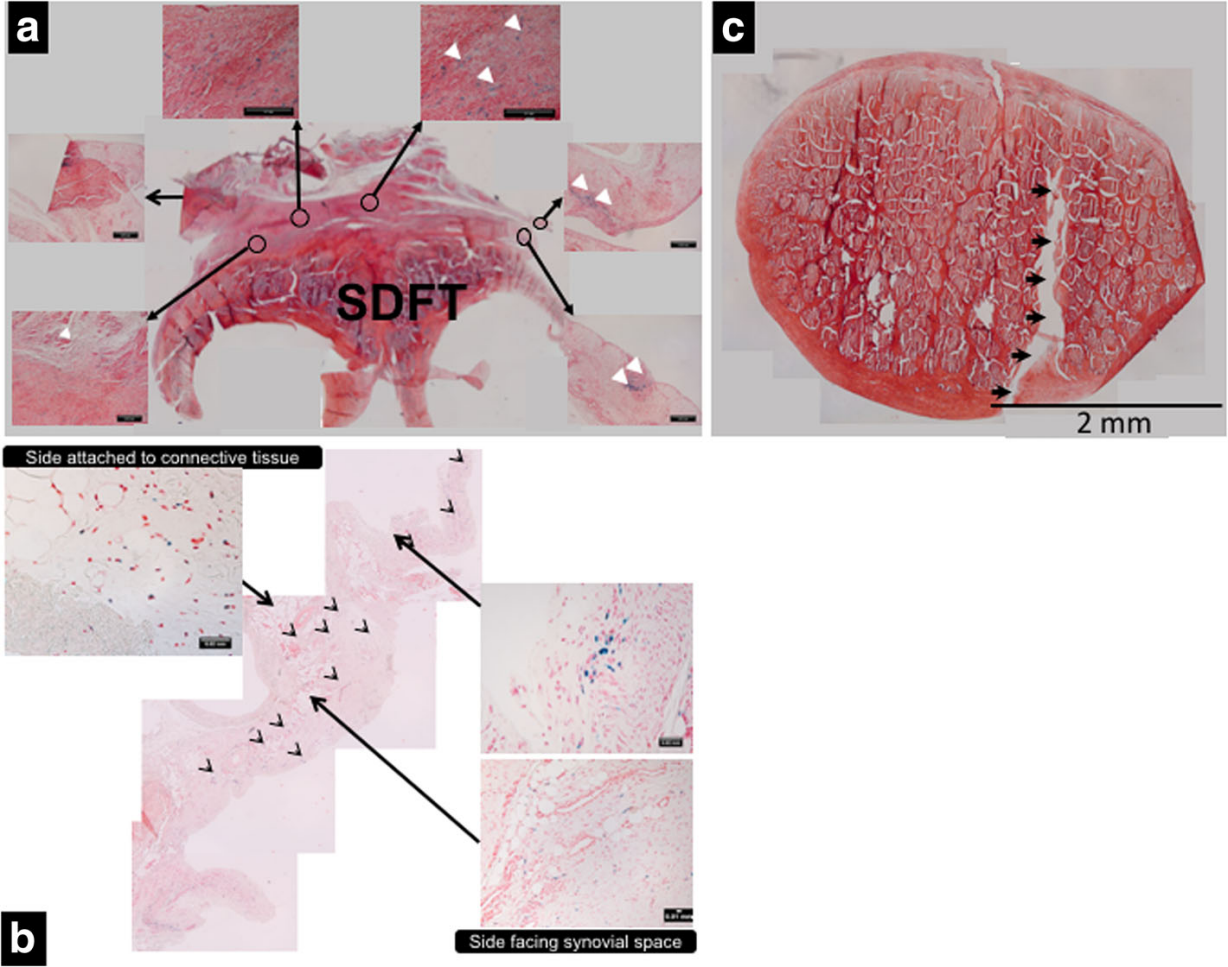
Montage representing whole section of SDFT bifurcation with dorsal synovium (H&E), with higher magnification showing presence of Prussian blue positive cells (white arrowheads sowing some examples) (**a**) which were also detected at different locations within the ventral synovial tissues (open arrowheads and blue stained cells in high power images) (**b**). Labelled cells were not detected in contra-lateral forelimb nor in PBS-injected tendons. Labelled cells not were detected within DDFT or lesion (**c**). These observations were consistent in all sheep implanted with cells. Arrows shown along length of lesion in (**c**). SDFT superficial digital flexor tendon

Histological examination of sheep treated with MION-labelled MSCs indicated Prussian blue (to stain for iron) positive cells within the palmar synovial tissue (segment J in Fig. 1e) on the palmar surface of the SDFT (Fig. 6a) and the dorsal synovial tissue (segment K in Fig. 1d) constituting the opposing dorsal synovial tissue to the DDFT (Fig. 6b). However, labelled cells were neither within the synovial covering of the deep digital flexor tendon nor in the tendon lesion (Fig. 6c). The Prussian blue positive cells were detected as non-homogeneous clusters engrafted within the synovium and distinct from iron associated with red cells within blood vessels (Fig. 6a, b).

**Fig. 7:**
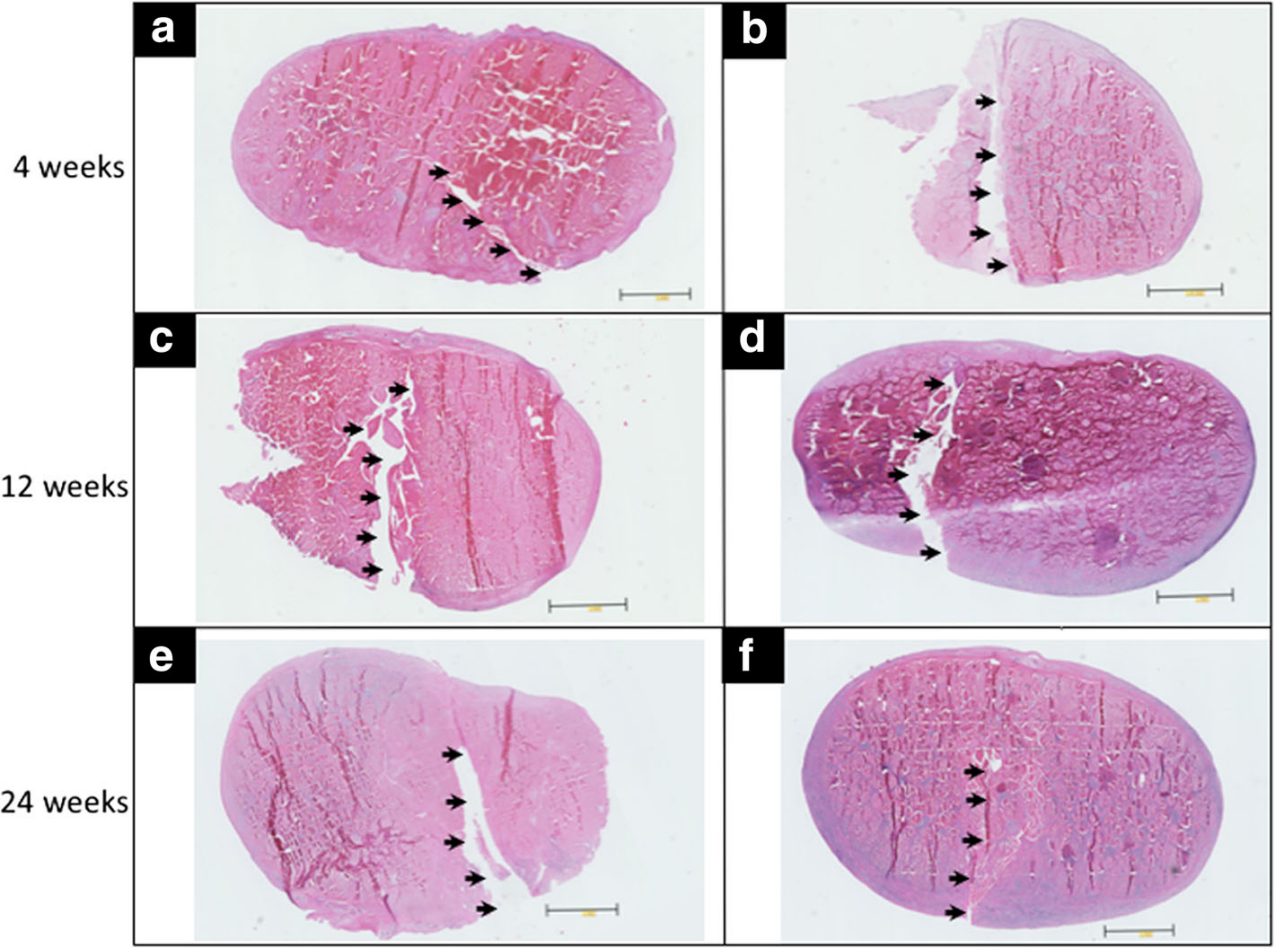
Images of transverse DDFT tendon sections of PBS-treated (**a, c, e**) and MSC-treated (**b, d, f**) sheep at 4, 12 and 24 weeks post implantation, respectively. Lesion persisted in all samples at 4 weeks (**a** versus **b**), 12 weeks (**c** versus **d**) and 24 weeks (**e** versus **f**). Arrow shown along length of lesion. Scale bar = 1 mm

Histological evaluation of transverse sections of the deep digital flexor tendon in sheep treated with non-labelled MSCs showed the persistence of the tendon lesion in all groups at 4, 12 and 24 weeks (Fig. 7a–g). These included those tendons which appeared closed on gross examination (Fig. 5c), indicating an absence of tendon healing at 4, 12 and 24 weeks post implantation.

### Engraftment of MSCs with the synovium by flow cytometry

**Fig. 8:**
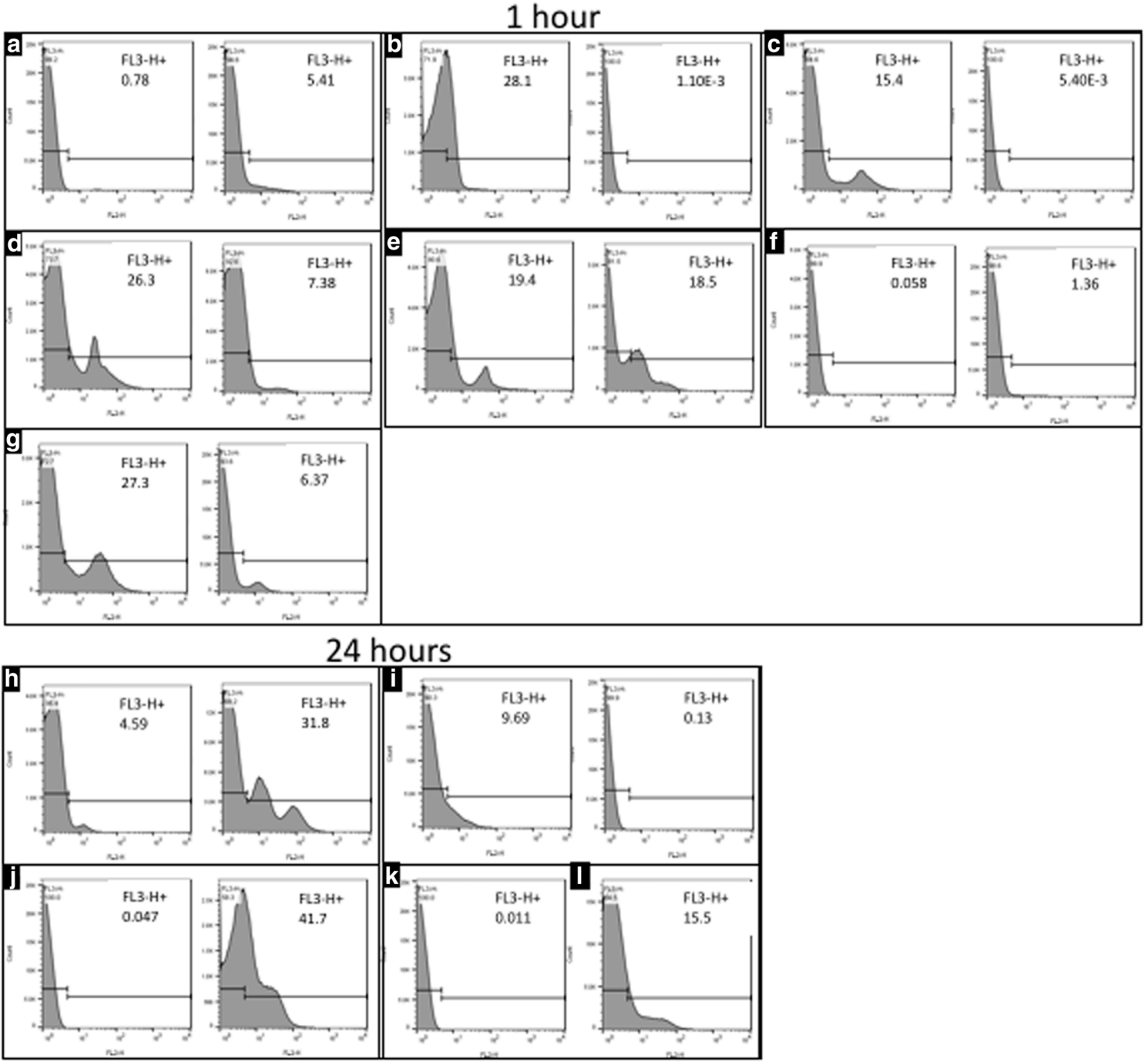
Flow cytometry graphs of MION-labelled MSCs isolated from tissue segments from two sheep at 1 h (**a–g**) and 24 h (**h–l**) after implantation. Tissue segments were dissected and adherent cells released by mild enzymatic digestion. **a, k** Distal lateral DDFT (segment A). **b** Distal medial DDFT (segment B). **c**, **j** Distal lateral SDFT (segment F). **d**, **l** Distal medial SDFT (segment G). **e**, **h** SDFT bifurcation (segment J). **f** Ventral synovium (segment K). **g**, **i** Sesamoid bones, distal to segment K. MION-labelled cells were detectable in segment J of both sheep at 1 h (**e**) and 24 h (**h**) post implantation. Cells were detectable at the level of proximal sesamoid bones (distal to segment K) in both samples at 1 h (**g**) but not at 24 h (**i**) post implantation. DDFT segments did not display MION-related fluorescence at either time point in both samples nor where cells were recovered from the synovial cavity (sample L). Sample identities are elaborated in Fig. 1 accompanying table

The number of cells isolated as a result of whole tissue digestion ranged from 4000 to 25,000 cells per segment. The cells isolated from sheep implanted for 1 h showed an absence of MION-related fluorescence in the lateral and medial DDFT segments A and B (Fig. 8a, b). MION-related fluorescence was detected in cells isolated from only one of the two sheep for lateral and medial SDFT segments F and G after 1 h (Fig. 8c, d). However, both sheep displayed MION fluorescence in cells isolated from SDFT bifurcation (Fig. 8e) and at the level of the sesamoid bones (Fig. 8g) after 1 h. Cells isolated after 24-h implantation displayed MION fluorescence at the tendon bifurcation site in both sheep (Fig. 8h) but not in the DDFT and SDFT segments and sesamoids (Fig. 8i–l). The percentage of cell retention was not quantified since only the outer surfaces of the tissue samples were digested to release cells adhered on the outside.

## Discussion

The present study sought to evaluate the distribution and localisation of MSCs following implantation within the digital tendon synovial sheath and their ability to heal an experimentally induced tendon lesion. The use of a hook knife with a fixed length and the naturally occurring gap in the overlying SDFT resulted in a highly reproducible intra-synovial surface lesion with prolapsed fibres that resembled naturally occurring tendon tears. The use of minimally invasive surgery in this model produced minimal inflammation and consequently low morbidity in the sheep, as evidenced by the lack of lameness and synovial effusion. Most interestingly, the created lesion failed to heal, even after 6 months, which is unusual in experimental surgical models but is a characteristic of natural intra-synovial tendon disease where lesions remain unaltered for many months. Thus, this study has demonstrated a novel model that reproduces many of the features of naturally occurring intra-synovial tendon disease. The absence of significant surgical trauma associated with this disease model makes this model particularly appropriate for investigating novel treatment strategies for intra-synovial tendon disease, such as rotator cuff tears.

The MSCs were isolated based on whole bone marrow plating, which is a relatively recent method applied for MSC recovery and expansion compared to the widely accepted method of Ficoll-based density centrifugation. Our previous work has shown that whole bone marrow plating could yield cultures with almost uniform positive staining for MSC-related antigens, although a small number of CD34 and CD45 positive cells were detectable by flow cytometry [40]. This method was an easier and reliable way to isolate MSCs and was therefore used for this study. MION labelling was not found to affect MSC characteristics including colony forming ability, proliferation and trilineage differentiation ability.

MIONS were used in an attempt to track the location of MSCs on MRI. MIONs contain iron and as this potentially may affect MSC behaviour we attempted to evaluate this in vitro, and were able to show no significant effect of population doubling or adipogenic and chondrogenic differentiation. However, osteogenic differentiation did appear to be adversely affected and this may be due to the presence of intracellular iron which has recently been shown to inhibit osteogenesis [42]. For this reason, the assessment of healing was done at the longer time points with unlabelled cells.

MIONs are a negative contrast material, resulting in signal void (hypointensity) in MRI. Gradient echo sequences were chosen in order to maximise the sensitivity to this artefact, and dual echo was used in order to specifically differentiate susceptibility artefacts from other causes of signal void (such as scar tissue). The main limit of this method is the difficulty to detect a hypointense signal void created by MIONs against the predominantly hypointense background signal of tendons due to the alignment of water molecules with the collagen. In order to track cells in tendons, the use of positive contrast material may be necessary or the magic angle artefact should be used [43], but specificity could be limited as tears can also result in increased signal within the tendons. Evaluation of quantitative methods, such as T2 or T1 mapping, was beyond the scope of this study. The analysis showed a non-generalised distribution of cells throughout the sheath with higher concentration in the region of tendon sheath cavity and synovial lining. The histology corroborated MRI findings, with Prussian blue positive cells identified in the synovial tissue sections from all implanted animals. Interestingly, the lesion was not visible on MRI as a result of the limited resolution of the MRI and the presence of hyperintense foci, most likely representing interfascicular matrix, visible in all tendons including non-injected control limbs, further confirming the lack of inflammation within the tendon as a result of the surgical lesion.

The implanted MSCs did not appear to home to the tendon lesion based on both MRI and histology of MION-labelled MSCs, which explains the lack of a detectable beneficial effect on healing at the later time points. This is in contrast to lesions created in the core of the sheep DDFT within the extra-synovial portion where retention of MSCs can be observed [44]. Interestingly, in our intra-synovial model, cells had adhered as clusters at specific sites within the synovial membrane tissue, suggesting that implanted MSCs attach to specific preferential stem cell niches within the synovium. It is not clear what factors enable the MSCs to engraft into this location, nor why they appear as clusters, although the presence of receptors on the synovial membrane and subsequent proliferation in situ after engraftment remain the most likely explanations. This adherence appears to be rapid since no labelled cells were recovered from the swabs taken on the synovial fluid 1 h after implantation. The use of pre-differentiated MSCs into a tendon progenitor cell may overcome the limitation to home into the tendon but this will need to be tested in the future. Although a dose response to applied MSCs was not performed in this study, it is possible that higher doses of cells may have promoted homing or closure of the lesion. However, given the rapid dispersion of the cells after injection and the absence of cells in either the SDFT or the DDFT, we propose that cell retention rather than higher doses may be more effective.

## Conclusions

The use of minimally invasive techniques in this model produces a non-healing intra-synovial tendon lesion in a tendon under compression which more accurately mimics the clinical situation of rotator cuff disease in humans. Furthermore, this model is associated with minimal morbidity and is ideal for evaluating novel biological therapies for augmenting intra-synovial tendon repair. However, intra-synovially administered bone marrow-derived MSCs did not appear to home to a tendon lesion and did not promote healing. We therefore hypothesise that physical or biological techniques to localise MSCs to or within the tendon lesion will be necessary to enable any therapeutically beneficial effect of MSCs.

## Abbreviations

DDFT: Deep digital flexor tendon
DMEM: Dulbecco’s modified Eagle’s medium
FBS: Foetal bone serum
H&E: Haematoxylin and eosin
IBMX: 3-Isobutyl-1-methyl xanthine
ITS+: Insulin–transferrin–selenium
MION: Magnetic iron-oxide nanoparticle
MRI: Magnetic resonance imaging
MSC: Mesenchymal stem cell
PBS: Phosphate buffered saline
SDFT: Superficial digital flexor tendon
